# Magnetic Molecularly Imprinted Nano-Conjugates for Effective Extraction of Food Components—A Model Study of Tyramine Determination in Craft Beers

**DOI:** 10.3390/ijms22179560

**Published:** 2021-09-03

**Authors:** Piotr Luliński, Marta Janczura, Monika Sobiech, Joanna Giebułtowicz

**Affiliations:** 1 Department of Organic Chemistry, Faculty of Pharmacy, Medical University of Warsaw, Banacha 1, 02-097 Warsaw, Poland; piotr.lulinski@wum.edu.pl (P.L.); mjanczura@wum.edu.pl (M.J.); 2 Department of Bioanalysis and Drugs Analysis, Faculty of Pharmacy, Medical University of Warsaw, Banacha 1, 02-097 Warsaw, Poland; joanna.giebultowicz@wum.edu.pl

**Keywords:** magnetic molecularly imprinted polymers, dispersive solid phase extraction, tyramine, craft beer, biogenic amine, liquid chromatography coupled with tandem mass spectrometry

## Abstract

In this paper, magnetic molecularly imprinted nano-conjugates were synthesized to serve as selective sorbents in a model study of tyramine determination in craft beer samples. The molecularly imprinted sorbent was characterized in terms of morphology, structure, and composition. The magnetic dispersive solid phase extraction protocol was developed and combined with liquid chromatography coupled with mass spectrometry to determine tyramine. Ten samples of craft beers were analyzed using a validated method, revealing tyramine concentrations in the range between 0.303 and 126.5 mg L^−1^. Tyramine limits of detection and quantification were 0.033 mg L^−1^ and 0.075 mg L^−1^, respectively. Therefore, the fabricated molecularly imprinted magnetic nano-conjugates with a fast magnetic responsivity and desirable adsorption performance could be an effective tool for monitoring tyramine levels in beverages.

## 1. Introduction

Tyramine (2-(4-hydroxyphenyl)ethylamine), a low molecular weight biogenic amine, causes serious toxicological effects in humans due to neurological or cardiovascular activity such as hypertensive crisis, severe headache, intracranial hemorrhage, neuronal sequelae, cardiac failure, or pulmonary edema [1]. The mammalian detoxification mechanism requires the monoamine oxidase system to metabolize tyramine, but this process could be insufficient in cases of inappropriate diet or an administration of selected drugs. The intracorporeal concentration of tyramine may increase after the consumption of tyramine-rich food and beverages. Tyramine is formed by conversion of tyrosine, the compound naturally occurring in wort while the beer manufacturing process is proceeded. The drinking of beer could be particularly risky due to high volumes and the frequency of consumption because of the inhibition effect of ethanol on the monoamine oxidase system, resulting in lower tyramine detoxification [2]. In a broad analysis made by the European Food Safety Authority (EFSA) in 2011, tyramine average occurrence value in beer was found to be 6.1 mg kg^−1^, and the maximum value was 24.7 mg kg^−1^. According to tyramine concentration in beer and beer consumption data, the analysis showed that tyramine exposure per day of beer consumption was in range of 18.5–124.6 mg day^−1^ (high beer consumption per day varied from 750–5040 g, typical ABV (alcohol by volume) of beer varied from 4 to 7%, but we can find beers with ABV from 0.5 to 20% or more). Despite the fact that, for healthy individuals, the minimal tyramine amount that caused toxic effect was 600 mg in a meal, the patients treated by classical monoamine oxidase inhibitors need only 6 mg of tyramine in a meal to observe adverse reactions connected with tyramine activity [3,4].

The concentration of tyramine in beer strongly depends on the quality of the raw material and brewing technique, but higher levels can be observed because of microbial contamination, or as a result of inappropriate storage, particularly of under-pasteurized beers which were contaminated with various strains of lactic acid bacteria [5,6,7]. A beer production process that avoids pasteurization and micro-filtration is frequently applied by independent producers to obtain high quality artisanal beers in various microbreweries. Craft beer is a very popular beverage due to its unique flavor and aroma that arises from a variety of recipes and a wide range of unconventional ingredients and alternative technological methods [8,9,10]. Nevertheless, careless selection of the brewery’s microbial starters and problems with good manufacturing and storage practices could counter the growth of *Lactobacillus brevis*, the best known tyramine producer in beer, and enhance the probability of increased levels of tyramine [11]. Taking into account dietary and food safety reasons, the determination of tyramine in beer specimens manufactured by microbreweries could have an important scope. 

The analysis of tyramine in beer is complicated due to the complexity of the sample [12]. The intrinsic variability of beers is a consequence of a great number of volatile compounds. However, a largely diverse group of organic molecules makes the analysis process challenging [13]. In order to determine tyramine, pre- or post-column derivatization reactions were employed prior to the instrumental detection. Nonetheless, those analytical methods suffered from time-consuming protocols affected by the derivatization reaction, stability, solubility, and compatibility of the derivatizing agents [14,15,16,17,18]. The derivatization step was not included only in a few tyramine quantification methods in beverages [19]. Application of mass spectrometry (MS) detectors with capillary electrophoresis as well as liquid (LC) or gas chromatography (GC) is an alternative for tyramine analysis [20,21,22]. However, to obtain optimal determination conditions, the pretreatment clean-up is necessary. Here, liquid–liquid or solid phase extractions (SPE) are versatile tools. Miao et al. [22] described solid phase “on-situ” quadraplex isotope dimethyl labeling for the analysis of biogenic amines in beers by LC-high resolution MS involving magnetic material. It allowed for the operational simplicity and rapidity of separation of magnetite in the external magnetic field as well as high extraction efficiency, the ability to extract analytes from large volume samples, and cost-effectiveness derived from the dispersive solid phase extraction (d-SPE) technique. In order to facilitate the separation process, the magnetite core was employed to commercial sorbents. Nevertheless, low selectivity and insufficient recoveries were noted for modified magnetized sorbents, hampering their utility. To overcome existing problems, new analytical strategies that allow for the lowering of the limits of quantification (LOQs), improved accuracy, enhanced selectivity, and minimized matrix effects (MEs), are highly required. Here, molecularly imprinted polymers (MIPs) could be recognized as valuable selective sorbents because of their synthetic process, which proceeds in the presence of the template molecule [23,24,25,26]. However, to elaborate the analytical method available for a more complex matrix, as well as to facilitate the process and make them faster, new strategies have been considered. One of the excellent tools used to fulfill the above mentioned demands is to merge MIPs with magnetic susceptible materials in order to obtain advanced core-shell imprinted nano-conjugates [27]. This allowed to combine the advantages of a high selectivity of imprinted sorbent with the operational facility from magnetic-susceptible materials. Apart of a few electrochemical methods devoted to the analysis of tyramine on imprinted materials [28,29,30], the magnetic dispersive molecularly imprinted solid phase extraction (m-d-MISPE) was not used in the analysis of tyramine in a complex matrix sample such as beer.

According to the available data, as high as 30% of the population declared consumption of craft beers in Poland [9]. Słomkowska and Ambroziak [31] described the biogenic amine profile of the most popular but not craft Polish beers, and tyramine was found in every of twenty-six samples, with a concentration of between 0.45 and 4.00 mg L^−1^. It should be underlined that the analysis was carried out at a time in which the overall diversity of craft beers on the market was limited.

In this paper, we were interested in fabricating an effective magnetic molecularly imprinted nano-conjugate, serving as a sorbent in the analytical strategy for the determination of food components, viz., beverages. In order to verify the hypothesis of whether magnetic molecularly imprinted sorbents could be a versatile tool in the determination of beer ingredients, a comprehensive characterization on the molecular level was provided, allowing us to confirm high specificity and structural properties of the sorbent. A model study of tyramine analysis in beer samples was carried out, involving m-d-MISPE combined with liquid chromatography, coupled with tandem mass spectrometry. The SPE process was analyzed, and the analytical method was validated. In order to show the applicability of new sorbent, the levels of tyramine in various Polish craft beers were analyzed.

## 2. Results and Discussion

### 2.1. Characterization of Magnetic Sorbent

**Figure 1:**
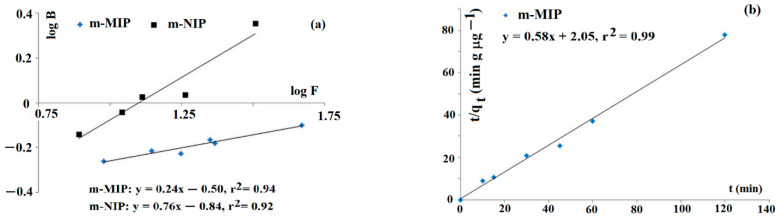
Freundlich isotherms for tyramine on m-MIP and m-NIP (**a**) and kinetic of adsorption on m-MIP (**b**).

Firstly, the characterization of the adsorption sites was carried out. The adsorption studies of imprinted Fe_3_O_4_@SiO_2_-MPS@MIP (coded as m-MIP) and non-imprinted Fe_3_O_4_@SiO_2_-MPS@NIP (coded as m-NIP) towards tyramine were carried out to confirm the heterogeneity of adsorption sites. The Freundlich model was employed to fit data. This model is suitable for adsorption in low concentration regions. The straight lines of log *B* versus log *F* are evidence that adsorption could be described by the Freundlich equation (Figure 1a). The estimated values of *m* for m-MIP and m-NIP were 0.24 and 0.76, respectively. The results indicated that m-MIP had a greater heterogeneous population of adsorption sites with respect to m-NIP (the heterogeneity increased as the value of m decreased).

The kinetics of adsorption could affect the sorption behavior of the sorbent in m-d-SPE. Thus, the kinetic of tyramine adsorption of m-MIP, employing the Ho–McKay model, was analyzed (Figure 1b). The linear function of *t*/*q_t_* against *t* was obtained, and the calculated values of *k*_2_ and *q*_e_ were as follows: *k*_2_ = 0.164 g µg^−1^ min^−1^ and *q*_e_ = 1.72 µg g^−1^, respectively.

**Figure 2:**
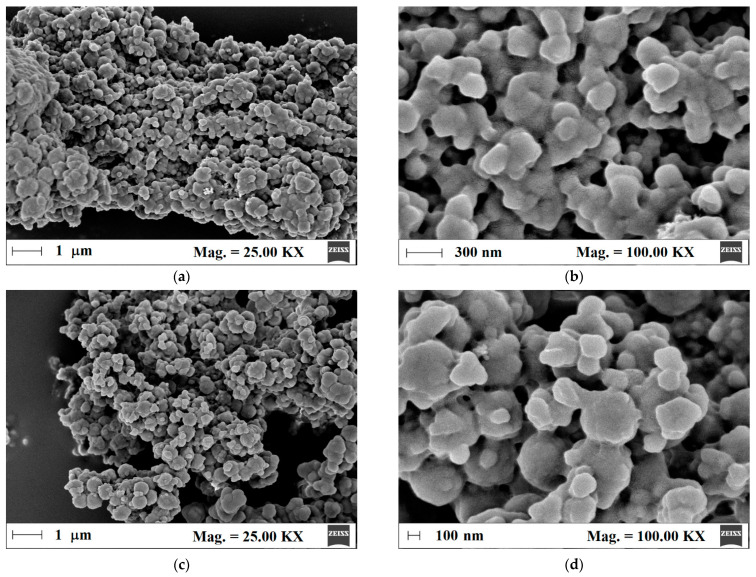
Micrographs of m-MIP (**a**,**b**) and m-NIP (**c**,**d**).

Next, the surface morphology of m-MIP and m-NIP was analyzed to prove the proceeding of polymeric shell on the magnetite core. Thus, for such purpose, the field emission scanning electron microscopy (FE-SEM) was employed (Figure 2a–d).

The morphologies of m-MIP and m-NIP were similar. The particles consisted of agglomerated entities with a diameter between 200 and 400 nm. The higher magnification micrographs revealed that spherical entities formed bigger structures that were uniformly coated by the organic polymeric layer.

**Figure 3:**
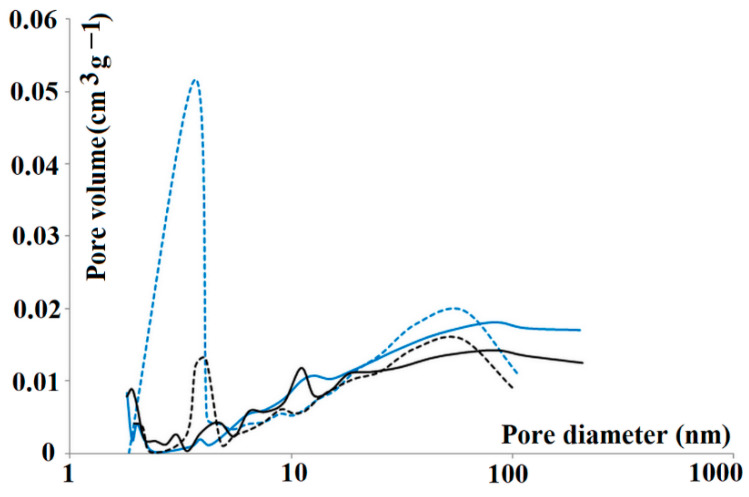
Pore size distributions on adsorption (solid lines) and desorption (dashed lines) branches of isotherms of all tested materials: m-MIP (blue) and m-NIP (black).

In order to explore the pore structure in detail, nitrogen sorption analysis was carried out. The nitrogen adsorption isotherms were used for both m-MIP and m-NIP to analyze the total specific surface area (Brunauer–Emmett–Teller isotherm) together with the cumulative surface area of pores (Barrett–Joyner–Halenda model) and the volume and area of the micropores (Harkins–Jura equation). The specific surface area of m-NIP was slightly higher with respect to m-MIP (14.31 and 12.41 m^2^ g^−1^, respectively). The micropore area and the external surface area of m-NIP were also higher than those of m-MIP (6.33, 7.98 m^2^ g^−1^, respectively, for m-NIP and 5.33, 6.88 m^2^ g^−1^, respectively, for m-MIP). Interestingly, the cumulative volumes of pores between 1.7 and 300 nm were higher for m-MIP than m-NIP, revealing an important difference between both materials. The adsorption/desorption cumulative volumes of pores were as follows: 0.0245 to 0.0237 cm^3^ g^−1^ and 0.0212 to 0.0194 cm^3^ g^−1^ for m-MIP and m-NIP, respectively. Finally, one of the parameters considered as being related to the imprinting process is the average pore size in the adsorption and desorption processes [32]. Pore size distributions on adsorption and desorption branches of isotherms of m-MIP and m-NIP are presented in Figure 3.

The adsorption and desorption average pore diameter were equal to 22.78 and 15.44 nm for m-MIP, respectively, and were significantly different from m-NIP, with respective values equal to 17.18 and 16.09 nm. It should be underlined that the difference was more evident for m-MIP. Smaller average pore diameters in the desorption branch of the isotherm than in the adsorption branch could prove the presence of bottle-shaped pores. The results indicated that both materials possessed different pore systems, confirming the effect of the template on the morphology of material [33].

**Figure 4:**
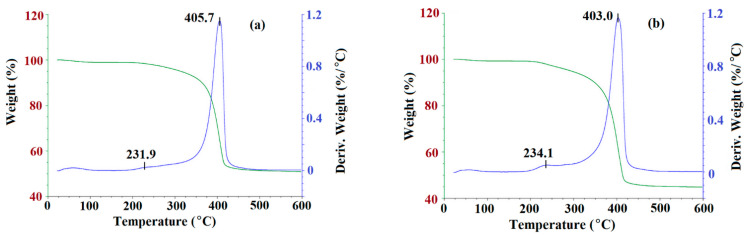
TGA curves of m-MIP (**a**) and m-NIP (**b**): Green line presents the loss of weight as the function of temperature; blue line is a derivative of weight loss as the function of temperature.

Next, the thermogravimetry analysis (TGA) was utilized to observe the degradation of m-MIP and m-NIP as a function of temperature. The TGA curves are presented in Figure 4. 

As can be seen, the decomposition patterns for m-MIP and m-NIP were very similar (Figure 4). The process started at about 210 °C and continued until 450 °C. The decomposition step consisted of two stages. First, a maximum of weight loss was observed at 231.9 °C and 234.1 °C for m-MIP and m-NIP, with the loss of 4% of total mass. Second, a maximum was observed at 405.7 °C and at 403.0 °C for m-MIP and m-NIP, respectively, with the loss of the initial mass of material being equal to 49.1% for m-MIP and to 55.1% for m-NIP. It could be supposed that the initial decomposition was attributed to the short chain degradation, as well as to the decarboxylation process, which was also responsible for stable decomposition in the range of 300–450 °C. It should be underlined that, in the decomposition process, the organic polymeric layer derived from methacrylates (methacrylic acid (MAA), ethylene glycol dimethacrylate (EGDMA), and 3-(trimethoxysilyl)propyl methacrylate (MPS)) was involved. The short maximum of weight loss at 50–55 °C could be explained by the loss of intrinsically bound water. The results proved that 50.9% of the m-MIP and 44.9% of m-NIP consisted of non-decomposed components. Iron and silicon oxides have melting points above 1000 °C, and both were highly stable in the studied temperature range: 25–600 °C [34].

**Figure 5:**
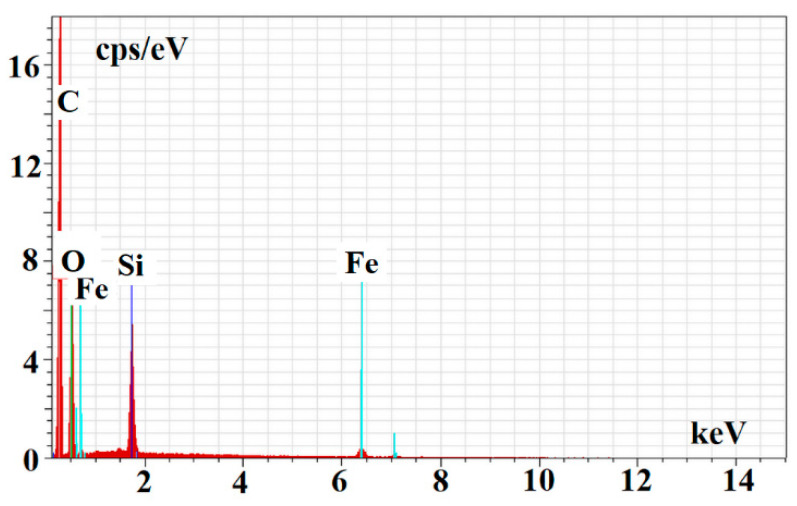
EDS spectrum of m-MIP.

Finally, the energy dispersive spectroscopy (EDS) was used to confirm the structural composition of m-MIP and m-NIP. Figure 5 presents EDS spectrum for m-MIP. 

The EDS analysis confirmed the proceeding of the polymer layer on the magnetite core. The following values of %wt of C, O, Si, and Fe were obtained for m-MIP: 51.14 ± 7.44, 32.58 ± 4.74, 9.80 ± 1.43, and 6.48 ± 0.94, and for m-NIP: 56.35 ± 8.19, 26.96 ± 3.92, 10.06 ± 1.46, and 6.63 ± 0.96, respectively.

**Figure 6:**
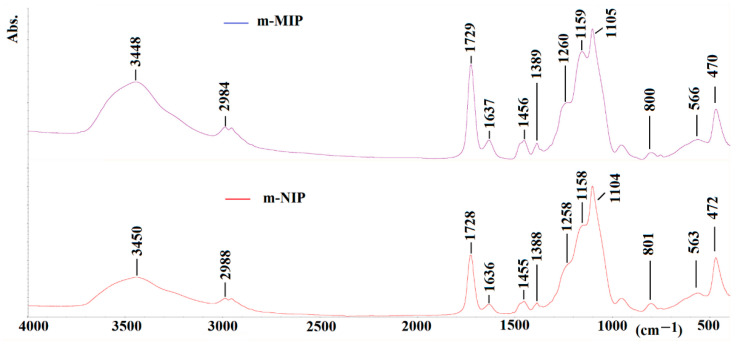
FTIR spectra of m-MIP (purple) and m-NIP (red).

Finally, the infrared (FTIR) analysis was employed to prove the structure of the obtained materials. The spectra of m-MIP and m-NIP are presented in Figure 6.

The characteristic vibration peaks derived from structural fragments of the polymer network can be seen. The assigning of peaks for m-MIP is as follow (value for m-NIP is presented in brackets): 3448 (3450) cm^−1^ for the –OH stretching vibration (broad); 2989 (2988) cm^−1^ for the –CH_2_– or –CH_3_ stretching vibration; 1729 (1728) cm^−1^ for the –C=O stretching vibration; 1637 (1636) cm^−1^ for the stretching of C=C bonds; 1456 (1455) cm^−1^ for the stretching of CH_2_–CH_2_; 1389 (1388) cm^−1^ for the stretching of –CH_3_; 1260 (1258) cm^−1^ and 1159 (1158) cm^−1^ for the asymmetric elongation of C–O–C bonds; 1105 (1104) cm^−1^ for the Si-O vibrations; 800 (801) cm^−1^ for the vibrations of Si-O-Si; 470 (472) cm^−1^ and 566 (563) cm^−1^ for the vibrations of Fe-O.

To sum up, it could be stated that the results from the morphological and structural analyses confirmed the presence of magnetic core in the material, as well as proceeding the organic polymeric shell. The adsorption studies proved that both tested materials presented different sorption characteristics, confirming the effect of the template on the morphology of m-MIP.

### 2.2. Analysis of Magnetic Dispersive Molecularly Imprinted Solid Phase Extraction

The analysis of the m-d-MISPE protocol is significant for the characterization of recognition ability of m-MIP. The suitable solvent used in the washing and eluting steps of the m-d-MISPE procedure should encourage the interaction to develop between the analyte and the monomer residues existing in the cavities, but should eliminate non-specific adsorption on the surface of polymer outside the cavities. Therefore, in this part, the impact of washing and elution solvents was analyzed. The following solvents in elution step were examined: 1% ammonium hydroxide in methanol, 1% formic acid in methanol, methanol, 40 mmol L^−1^ aqueous acetate ammonium in methanol, 1% ammonium hydroxide in water, and 1% formic acid in water. Then, the following solvents in the washing step were tested: 0.01% ammonium hydroxide in water, water, 15% methanol in water, or 0.01% formic acid in water.

**Figure A1:**
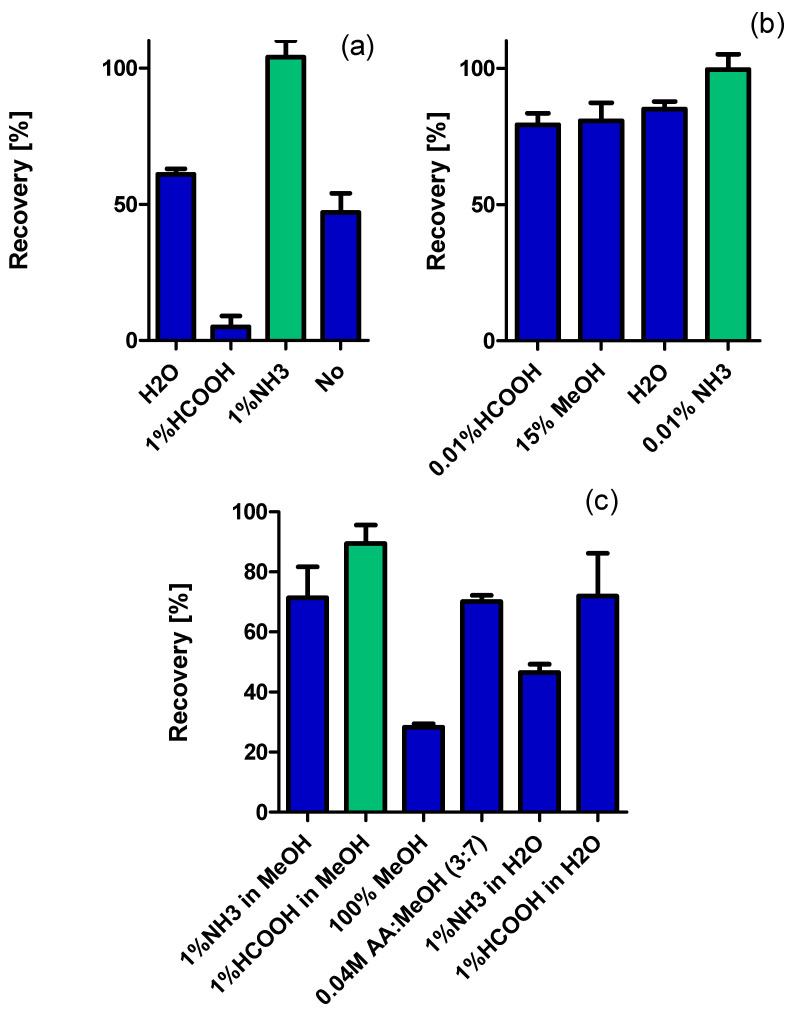
Recoveries of tyramine in analysis of m-d-SPE process: loading (**a**), washing (**b**) and eluting (**c**) steps.

The highest recovery was observed when 1% of the formic acid in methanol was used as an eluent and a volume of 0.75 mL was sufficient to desorb all adsorbed analyte effectively. The lowest analyte loss was due to washing the sorbent with 0.01% ammonium hydroxide in water (Figure A1 in Appendix A). Simultaneously, no ME was observed (ME = 97–102%) for either of tested solvents, proving the high purity of the extract. 

Next, the loading and elution time were tested. The sample contact time was set at 5, 10, 15, and 30 min. No significant differences (<2%) in adsorption between 5 and 30 min were observed. Thus, a 5 min contact time for loading was selected as optimal. The optimal elution time was 10 min, being 6% higher than after 5 min.

The impact of the dilution of the beer sample on recovery of tyramine was also analyzed. The recovery was significantly higher when 1% ammonium hydroxide was used to dilute sample, compared to an undiluted sample or a sample diluted with water. 

### 2.3. Analytical Method Validation

**Figure 7:**
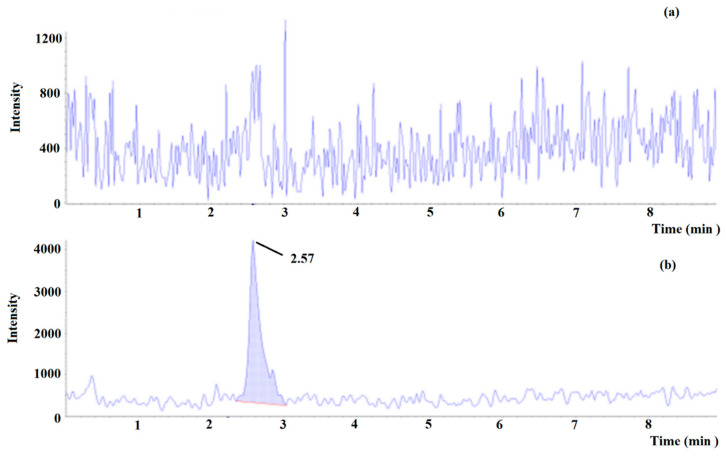
Multiple reaction monitoring (MRM) chromatogram of tyramine in (**a**) blank beer and (**b**) blank beer spiked at lower limit of quantitation (0.15 mg L^−1^).

The calibration curve obtained by the weighted linear regression analysis (*x*^−1^) was linear in the range between 0.15 and 75 mg L^−1^ (*r*^2^ ≥ 0.99), the limit of detection (LOD) of 0.033 mg L^−1^, and the LOQ of 0.075 mg L^−1^. The values of regression parameters (and their standard deviation), described by the equation: y = ax + b, were calculated as: a = 0.392 ± 0.023 and b = 0.0204 ± 0.0065. The mean recovery was 89%. The accuracy for the lower limit of quantification (LLOQ, 0.15 mg L^−1^) was 99% (RSD = 3.1%, *n* = 6, Figure 7). The accuracy and the intermediate precision for the quality control 1 (QC1, 3 mg L^−1^), QC2 (30 mg L^−1^) and QC3 (75 mg L^−1^) samples were 103% (RSD 4.9%, *n* = 6), 98% (RSD 1.7%, *n* = 6) and 104% (RSD 4.6%, *n* = 6), respectively. No ME for tyramine was observed, proving a high extract purity. The absolute ME ranged from 100% to 105%. The relative ME was equal to 1.0%. The extracts were stable up to 24 h in the autosampler (98% for QC1 and 101% for QC3). Satisfactory results of the dilution integrity test were obtained with an accuracy of 102% (RSD = 1.6%, *n* = 3). No carry-over was detected.

Finally, the validation parameters of the newly proposed analytical method were compared with other methods presented in literature and are summarized in Table 1. Most of the methods required sample derivatization with isobutyl chloroformate, dansyl chloride, o-phthaldialdehyde, diethyl ethoxymethylenemalonate, or 2,6-dimethyl-4-quinolinecarboxylic acid N-hydroxysuccinimide ester, which was not needed in the proposed one. The lowest concentration on the calibration curve of our method (0.15 mg L^−1^) was higher than in the methods developed by Miao et al. [22] (0.001 mg L^−1^), Almeida et al. [20] (0.01 mg L^−1^), Daniel et al. [21] (0.05 mg L^−1^), and He et al. [18] (0.14 mg L^−1^). The method precision (1.7–4.9%) was comparable to other methods (0.72–7.4%), except for the method developed by Angulo et al. [35], in which the precision was 11.5%. However, it should be highlighted that this validation data should be compared with caution, since most of the papers present only scarce data on their validation methodology, and only basic validation parameters were tested. Namely, no method was validated according to the FDA guideline.

### 2.4. Analysis of Tyramine in Beer Samples

Finally, the validated analytical method was applied for the determination of tyramine levels in selected beers available in Polish market. For that purpose, a set of ten different beers from one craft brewery was utilized. 

**Figure 8:**
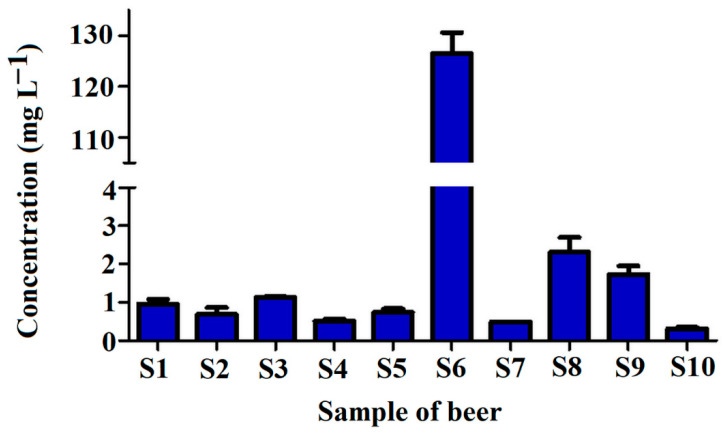
Concentration of tyramine in tested craft beer samples **S1**–**S10** (for sample composition please refer to Table 2).

The samples of each beer were collected just after the bottle was opened to analyze the level of tyramine in beer ready for consumption. The results showing the concentration of tyramine determined in analyzed beers are presented in Figure 8.

As can be seen, the concentration of tyramine in beer samples varied significantly. Mostly, the compound levels were in the range between 0.303 and 2.307 mg L^−1^, except for the sample S6, with a tyramine concentration as high as 126.5 mg L^−1^. The highest tyramine content in S6 is related to the presence of coffee (that contains tyramine intrinsically) in the sample [37].

The tyramine concentrations in all tested beers except the sample of S6 were very similar to the results presented in other studies [35,36]. For example, Miao et al. [22] found the tyramine levels in six various beers between 0.138 and 2.912 mg L^−1^; Tang et al. [17] found tyramine in eighteen beer samples at levels between 2.90 and 7.15 mg L^−1^; Almeida et al. [20] determined tyramine in beers in ranges between 0.394 and 5.916 mg L^−1^; Romero et al. [38] found tyramine concentration in Spanish beer samples between 0.26 and 31.69 mg L^−1^; and Słomkowska and Ambroziak [31] found tyramine levels between 0.48 and 4.00 mg L^−1^ in Polish (not craft) beers. The high tyramine level in the sample S6 could be explained by the composition of the beer, which consisted of chocolate wheat and coffee. Such a high level of tyramine was not detected in previously published studies. However, Kalac et al. [7] found a level of tyramine at 102 mg L^−1^ in one sample of bottom fermented pale lager from Czech Republic, and Lorencova et al. [39] described levels of tyramine in selected samples of tested Czech beers even up to 84.1 mg L^−1^. Similar results were obtained by Redruello et al. [16], finding in one sample of Spanish dark lager tyramine at a concentration of 58.3 mg L^−1^. 

## 3. Materials and Methods

### 3.1. Materials

2-(4-Methoxyphenyl)ethylamine (template), methacrylic acid (functional monomer, MAA), ethylene glycol dimethacrylate (cross-linker, EGDMA), tetraethoxysilane, 3-(trimethoxysilyl)propyl methacrylate (MPS) and 2,2′-azobis(2-methylpropionitrile) (initiator) were purchased from Sigma–Aldrich (Steinheim, Germany). Internal standard (IS) of 4-tyramine-*d*_4_-hydrochloride was bought from Toronto Research Chemicals (North York, ON, Canada). Trisodium citrate dehydrate, sodium hydroxide, sodium nitrate, ferrous sulphate heptahydrate, ammonium hydroxide, ethanol, anhydrous toluene, formic acid, ammonium acetate, acetonitrile were delivered from POCh (Gliwice, Poland). Ultrapure water was delivered from a Hydrolab HLP 5 system (Straszyn, Poland). 

Samples of craft beers were bought at local supermarkets. All below-mentioned samples were produced in one brewery manufacturing beer in Poland (Brewery Pinta, Wieprz, Poland). Only beers sold in glass bottles were purchased. The following specimens were analysed: A ja pale ale (coded as S1), Bawarka (coded as S2), Czarna Dziura (coded as S3), Pierwsza pomoc (coded as S4), Apetyt na życie (coded as S5), I’m so horny! (coded as S6), Dobry wieczór (coded as S7), Atak chmielu (coded as S8), Mini-maxi IPA (coded as S9), and Viva la Wita (coded as S10). All beers were composed of water. Detailed composition of analyzed specimens is presented in Table 2.

### 3.2. Synthesis of Sorbent

#### 3.2.1. Preparation of Functionalized Magnetic Core

The magnetic core was prepared [40] prior to functionalization by a silane derivative providing the functional groups enabling the polymerization of the imprinted layer on its surface. The synthetic details of core preparation are described in the Appendix A.

#### 3.2.2. Synthesis of Imprinted Polymeric Shell

The magnetic core-shell polymerization process was carried out to prepare imprinted Fe_3_O_4_@SiO_2_-MPS@MIP coded as m-MIP and non-imprinted Fe_3_O4@SiO_2_-MPS@NIP coded as m-NIP. The process was proceeded in the same way for MIPs and NIPs except in the synthesis of NIPs, where the addition of the template was omitted. 

In general, a mixture of 30.2 mg (0.2 mmol) of 2-(4-methoxyphenyl)ethylamine (template) and 68.9 mg (0.8 mmol) of MAA (functional monomer) were dissolved in 10 mL of toluene (solvent) and incubated for 24 h in the dark. Then, an amount of 287.3 mg of Fe_3_O_4_@SiO_2_-MPS was placed in a round-bottom flask together with 15 mL of toluene, 754 µL (4 mmol) of EGDMA, and 20 mg of 2,2′-azobis(2-methylpropionitrile). Then, the flask was put in an ultrasound bath for 5 min prior to purge with nitrogen for 5 min. After that, the flask was placed in a silicone oil bath on the magnetic stirrer and heated to 100 °C. After being left to react overnight, the mixture was transferred to small Berzelius beakers and rinsed with toluene (2 × 20 mL), methanol (2 × 20 mL), 40 mmol L^−1^ aqueous ammonium acetate—methanol 30:70 *v*/*v* (2 × 20 mL), and finally methanol (2 × 20 mL), all of which was done with the help of a magnet to separate the material. The template removal step was proceeded in the Soxhlet apparatus and lasted 36 h with a volume of 120 mL of methanol. To have a comparable treatment of the polymers, imprinted and non-imprinted polymers followed the same procedure.

**Table 2:** Detailed description of analyzed beers.

Code	Name	Style [41]	Blg ^a^	ABV ^b^	Composistion				
					Barley Malts	Wheat/Rye Malts	Hops	Yeast	Additions
S1	A ja pale ale	American Pale Ale	12°	5.0%	pale ale, Caraamber^®^	-	(United States): Columbus, Centennial, Cascade, Simcoe^®^, Citra^®^	SafAle^TM^ US-05	-
S2	Bawarka	Hefeweizen	13°	5.7%	pilsner, Carahell^®^	Wheat pale	(Germany): Mittlefruh	SafBrew^TM^ WB-06	-
S3	Czarna Dziura	Schwarzbier/Dark Lager	11.5° (ibu 38)	4.5%	Weyermann^®^ malts: pilsner, Munich (II), Carafa^®^ (III) Special, dyeing malt extract Sinamar^®^	-	(Germany): Tradition, Spalt Select	Saflager^TM^ W 34/70	-
S4	Pierwsza pomoc	Polish Light Pils (pale lager)	10.5°	4.1%	pilsner, Munich (II), Caramunic^®^ (II), Carapils^®^	-	(Poland): Marynka, Lubelski	bottom fermenting yeast: SafLager^TM^ W 34/70	-
S5	Apetyt na życie	Rye Beer	13.1° (ibu 18)	5.0%	Weyermann^®^ malts: pilsner, Vienna, roasted Carafa^®^ Special (I)	Weyermann^®^ rye malt, caramel rye Cararye^®^	(Germany): Tettnanger, Spalt Select	Safbrew^TM^ WB-06	-
S6	I’m so horny!	Espresso Lager	18°	6.7%	Weyermann^®^ malts: pilsner, Munich (I), Chit	Chocolate wheat malt	Styrian Golding (Slovenia)	Saflager^TM^ S-189	coffee: Adelante and Rio Azul (Guatemala)
S7	Dobry wieczór	Oatmeal Stout	13.5° (ibu 32)	4.5%	Weyermann^®^ malts: pale ale, Caramunich^®^ (II), Caraaroma^®^ (II), Carafa^®^ (I)	-	East Kent Golding (United Kingdom)	Safale^TM^ US-04	oat flakes
S8	Atak chmielu	American India Pale Ale	15°	6.1%	pale ale, melanoidin, Carared^®^, Carapils^®^	-	(United States): Citra^®^, Simcoe^®^, Cascade, Amarillo^®^	SafAle^TM^ US-05	-
S9	Mini-maxi ipa	Non-Alcoholic Session India Pale Ale	-	>0.5%	Pilsen, Carapils^®^	-	(United States): Citra^®^, Mosaic^®^	SafAle^TM^ LA-01	-
S10	Viva la Wita	Imperial Witbier	16.5°	5.7%	Weyermann^®^ malt: pilsner	Weyerman^®^ wheat malt	Styrian Goldings (Slovenia), Saaz (Czech Republic), Citra^®^, Palisade^®^ (United States)	Safbrew^TM^ S-33	non-malted wheat, spices: coriander, Curaçao peel, orange peel

^a^ Blg is extract and describes the amount of sugar in the wort. Blg determines how much sugar was in the beer before fermentation. ^b^ ABV: alcohol by volume.

### 3.3. Instruments

The analysis was performed using an Agilent 1260 Infinity system (Agilent Technologies, Santa Clara, CA, USA), equipped with a degasser (G4225A), an autosampler (G1367E), a thermostatted column compartment (G1316C), and a binary pump (G1312B) coupled to QTRAP 4000 hybrid triple quadrupole/linear ion trap mass spectrometer (AB Sciex, Framingham, MA, USA). The turbo ion spray source was operated in positive mode. The curtain gas, ion source gas 1, ion source gas 2, and collision gas (all high purity nitrogen) were set at 345 kPa, 207 kPa, 276 kPa, and “high” instrument units (4.6 × 10^−5^ Torr), respectively. The ion spray voltage and source temperature were 5000 V and 600 °C, respectively. The target compounds were analyzed in multiple reaction monitoring (MRM) mode. The quantitative MRM transitions, declustering potential (DP), and collision energy (CE) for tyramine and tyramine-*d*_4_ (internal standard, IS) were (*m*/*z*) 138/121 (DP = 16 V, CE = 13 V) and (*m*/*z*) 142/125 (DP = 51 V, CE = 15 V). Chromatographic separation was achieved with a Kinetex^®^ EVO C18 column (100 mm × 4.6 mm, 2.6 µm) from Phenomenex (Torrance, CA, USA). The column was maintained at 40 °C at a flow rate of 0.5 mL min^−1^. The mobile phases consisted of 20 mmol L^−1^ aqueous ammonium acetate as eluent A and acetonitrile, with 0.2% formic acid as eluent B. The gradient (%B) was as follows: 0 min, 10%; 1 min, 10%; 3 min, 95%; 5 min, 95%. The re-equilibration of the column to the initial conditions lasted 1.5 min. 

The surface morphology analysis using scanning electron microscopy (SEM) with a Merlin FE-SEM (Zeiss, Oberkochen, Germany) and the X-ray energy dispersive spectroscopy (EDS) analysis using an EDS X-ray detector (Brucker, Mannheim, Germany) were performed at the Faculty of Chemistry, University of Warsaw, Poland. The samples were Au/Pd sputter-coated before SEM analysis. The porosity data were determined using the adsorption isotherm of N_2_ at 77 K (BET) on an ASAP 2420 system (Micromeritics Inc., Norcross, GA, USA) at the Faculty of Chemistry, Maria Curie-Skłodowska University, Lublin, Poland. The infrared (FT-IR) spectra were recorded on Nicolet iS50 FT-IR (Thermo Fisher Scientific, Waltham, MA, USA) at the Biological and Chemical Research Centre, University of Warsaw, Poland. The thermogravimetry analyses (TGA) were performed at the Faculty of Chemistry, Warsaw University of Technology, Poland, on a Q600 thermogravimetric analyzer (TA Instruments, New Castle, DE, USA) in an argon atmosphere with heating rate 5 °C min^−1^.

### 3.4. Adsorption Studies

For isotherm analysis, polypropylene tubes were filled with 10 mg of m-MIP or m-NIP particles and a volume of 1 mL of different methanol–water (85:15 *v*/*v*) standard solutions of tyramine (concentrations between 10 and 50 μg L^−1^) were added. The tubes were sealed and oscillated by a shaker at room temperature for 2 h. Then, the tubes were centrifuged, and the aliquots of supernatant were used to analyze the unbound amounts of each compound by LC-MS/MS. For kinetics, the tubes were prepared as above but different times of oscillation were employed (5, 10, 15, 30, 45, 90, 120, and 180 min). Then, the tubes were treated in the same manner as described above. All measurements were carried out in triplicate. The binding capacities (*B*, μg g^−1^) of m-MIP or m-NIP were calculated according to Equation (1):

*B* = (*C_i_* − *C_f_*)*V*/*M* (1)

where *V* represents a volume of solution (L), *C_i_* represents the initial solution concentration (μg L^−1^), *C_f_* represents the solution concentration after adsorption (μg L^−1^), and *M* is the mass of particles (g). The adsorption isotherm was characterized using the Freundlich model presented in Equation (2):

(2) $$B=aF^{m}$$

where *a* is the measure of the capacity (*B_max_*), *m* is a heterogeneity index, and *F* is the concentration of the analyte in equilibrium state. The kinetic of adsorption was calculated using Ho–McKay model, according to Equation (3):

(3) $$\frac{t}{q_{t}}=\frac{1}{k_{2}{q_{e}}^{2}}+(\frac{1}{q_{e}})t$$

where *k*_2_ is the second-order-rate constant at the equilibrium (g µg^−1^ min^−1^), *q*_e_ is the adsorption capacity at equilibrium (µg g^−1^), *q*_t_ is the adsorption capacity at *t*, time (in min).

### 3.5. Analysis of Magnetic Dispersive Molecularly Imprinted Solid Phase Extraction

A beer sample (1 mL) diluted with either 1% of ammonium hydroxide (1:2, *v*/*v*) or with water or with 1% of formic acid or undiluted, was spiked with 500 µg L^−1^ of tyramine standard solution and loaded to 10 mg m-MIP in Eppendorf test tubes. Then, the tube was put on the vortex to provide contact time with the sorbent for 5 min (in the analysis of the loading time, the step lasted 5, 10, 15, and 30 min). Afterwards, the supernatant, separated from the sorbent by an external magnetic field, was discarded, and the washing with 1 mL of 0.01% ammonium hydroxide for 5 min was proceeded on the vortex (in the analysis of washing solvent, water, 15% methanol, and 0.01% formic acid were tested). The supernatant was likewise discarded as described above. Finally, the elution occurred by adding 750 µL of 1% formic acid in methanol (other solutions tested were as follows: 1% ammonium hydroxide in water and methanol, 1% formic acid in water, methanol, and 40 mmol L^−1^ aqueous ammonium acetate–methanol (30:70, *v*/*v*). The elution time was set for 10 min (in the analysis of the elution time, the step lasted 5, 10, 15, and 30 min). The elution fraction was separated from the sorbent by application of an external magnetic field, diluted with 40 mmol L^−1^ aqueous ammonium acetate (1:2, *v*/*v*) and injected to LC.

### 3.6. Method Validation

The method was validated for the accuracy, precision, LOD, LOQ, linearity, ME, dilution integrity, and stability of the extract. The validation was performed at level two (a single laboratory validation level) for a single matrix [42]. For that purpose, three different commercial brands of beers were used, as recommended by FDA. 

The calibration standards, LLOQ and QC samples were prepared by spiking blank beer with known quantity of analytes. The matrix blank was obtained by removal of tyramine from the beer using selective m-d-MISPE protocol. The blank purity was confirmed using LC-MS. The linearity range was selected between 0.15 and 75 mg L^−1^. Calibration curves (*n* = 3) were constructed by plotting peak area ratios of the targeted analyte to the area of IS versus the nominal concentration of the analyte. The accuracy and intermediate precision were determined using three matrix brands and were conducted during four separate runs (*n* = 6, two replicate samples per one beer brand) for LLOQ (0.15 mg L^−1^) and QC samples (3, 30 and 75 µg L^−1^). The LOD and LOQ were defined as a signal-to-noise ratio of 3 and 10, respectively. Carry-over was studied by placing a blank sample after calibration standard at the upper limit of quantification (ULOQ, 75 mg L^−1^). Dilution integrity was determined by spiking the matrix with an analyte concentration three times higher than the ULOQ (*n* = 3, 225 mg L^−1^) and diluting three times with water. The autosampler stability was determined after 24 h of extract storage in an autosampler (4 ± 0.5 °C). Various sources of beers (*n* = 3) were used for the evaluation of the ME. Due to the lack of a blank matrix, the calculations that were based on the slopes of the calibration curves were applied. The calibration curves were prepared by using beer samples and the solvent. The absolute ME was calculated based on a comparison of the slopes of the calibration curves obtained for beer samples (different brands) and solvent. Each sample was spiked with IS. The relative ME (adjusted to IS) for analytes was expressed as CV (%) of the slopes of the calibration curves. 

### 3.7. Beer Sample Preparation

A volume of 1 mL of sample from each beer (*n* = 10) was taken and examined immediately after the opening. Always three independent samples were analyzed. Beer samples were decarbonated by ultrasonic bath and centrifuged (5 min, 10,000 rpm). Then, each beer aliquot was diluted with 1% ammonium hydroxide (1:2, *v*/*v*) and shaken for 3 min on the vortex.

A volume of 0.1 mL (5 mg L^−1^) of IS was added to 0.9 mL of the diluted beer samples and mixed with 10 mg of m-MIP for 5 min. Afterwards, the supernatant, separated from the sorbent by an external magnetic field, was discarded, and the sorbent was washed with 1 mL 0.01% ammonium hydroxide in water for 5 min on the vortex. The supernatant was removed in the same manner as described above. Then, a volume of 0.75 mL of 1% formic acid in methanol was incubated with the sorbent for 10 min on a vortex. The elution fraction was separated from the sorbent by application of an external magnetic field, diluted with 40 mmol L^−1^ aqueous ammonium acetate (1:2, *v*/*v*) and injected to LC. 

## 4. Conclusions

A magnetic imprinted nano-conjugate core-shell material was elaborated and used as an effective sorbent in the magnetic dispersive solid phase extraction. The extraction process was combined with liquid chromatography coupled with tandem mass spectrometry analysis, providing an effective and versatile analytical strategy for beverage analysis. The structural characterization allowed us to confirm the fabrication of magnetite core functionalized by a molecularly imprinted shell. The morphology revealed an extension of surface of magnetic molecularly imprinted polymer, and the heterogeneous population of adsorption sites was confirmed using a Freundlich model. The method was validated, revealing satisfactory analytical performance for analysis of beverage samples. The analysis of craft beers from the Polish market revealed significant differences in the concentration of tyramine, depending on the composition of the sample. High tyramine level was found in one sample containing chocolate wheat and coffee.

Future studies should include a more comprehensive investigation into the beer brew process on different stages of the production to control the tyramine levels. Additionally, the monitoring of the tyramine level in components added to beers such as coffee or specimens should be considered.

## Figures and Tables

**Table 1:** Tyramine analyses in samples of beer by LC, GC, and CE using different extraction techniques and instrumental analyses.

Extraction Method	Instrumental Analysis	Derivatisation Reagent	Range (mg L^−1^)	LOD (mg L^−1^)	Precision (%) (QC Used) ^b^	IS	Ref.
DLLME	GC-MS	isobutyl chloroformate	0.010–15	0.007	5	hydroxyamphetamine	[20]
-	HPLC-UV	dansyl chloride	1–40	0.03	11.5 (5 mg L^−1^)	1,7-diaminoheptane	[35]
d-SPE (PVPP)	CE-MS	-	0.05–100	0.002	nd	1,7-diaminoheptane	[21]
LLE	CE-UV	-	0.14–21	0.08	3.1 ^a^ (3.4 mg L^−1^)	no	[18]
IL-UALLME	HPLC-FL	DMQ	0.5–50	0.005	3.5 (nd)	no	[14]
MSPE	HPLC-MS	-	0.001–5	0.00002	4.2 (0.5 mg L^−1^)	formaldehyde-*d*_2_ (dimethyl labeling)	[22]
-	HPLC-FL	o-phthaldialdehyde	0.15–5	0.053	5.7–7.4 (nd)	octylamine	[15]
-	HPLC-UV	DEEMM	0.62–206	0.12	0.72 ^a^ (27 mg L^−1^)	L-aminoadipic acid	[16]
d-SPE (PVPP)	HPLC-FL	dansyl chloride	0.5–20	0.02	2.5 (5 mg L^−1^)	diaminoheptane	[36]
m-d-MISPE	HPLC-MS	-	0.17–75	0.033	1.7–4.9 (3–75 mg L^−1^)	tyramine-*d*_4_	this study

^a^ RSD of the peak area; ^b^ Concentration used to calculate recovery; DEEMM: diethyl ethoxymethylenemalonate; DLLME: dispersive liquid–liquid microextraction; DMQ: 2,6-dimethyl-4-quinolinecarboxylic acid N-hydroxysuccinimide ester; IL-UALLME: ionic liquid-based ultrasound-assisted liquid–liquid microextraction; LLE: liquid–liquid extraction; m-d-MISPE: magnetic dispersive molecularly imprinted solid phase extraction; MSPE: magnetic solid phase extraction; nd: no data; PVPP: polyvinylpolypyrrolidone.

## Data Availability

The data presented in this study are available on request from the corresponding author.

## Appendix A. Synthesis of Magnetic Core and Functionalization

The Fe_3_O_4_ magnetic nanoparticles were synthetized by an addition of 2.94 g (10 mmol) of trisodium citrate dehydrate, 1.63 g (40 mmol) of sodium hydroxide, and 34.2 g (400 mmol) of sodium nitrate, which were dissolved in a volume of 180 mL of ultrapure water. The reaction was carried out in a round-bottom flask equipped with reflux and placed on a stirrer with heating plate (Heidolph, Germany). The mixture was stirred and heated to 100 °C until a clear solution was observed. A volume of 20 mL of 1 mol L^−1^ aqueous ferrous sulphate heptahydrate solution was added and was maintained at 100 °C for one hour. After letting it cool down slowly to room temperature, a dark brown precipitate was observed. It was separated by a magnet (discarding the solution) and, then, the particles were washed with ultrapure water for several times (20 mL, each). Then, the obtained Fe_3_O_4_ magnetic nanoparticles were dried at room temperature prior to the modification with tetraethoxysilane to obtain Fe_3_O_4_@SiO_2_. An amount of 600 mg of Fe_3_O_4_ was suspended in a volume of 80 mL of ethanol and 8 mL of ultrapure water under ultrasonication (Bandelin, Germany) for 15 min in a round-bottom flask. After that, a volume of 10 mL of ammonium hydroxide and a volume of 4 mL of tetraethoxysilane was added. The reaction was carried out for 12 h at room temperature with stirring. The product was separated with a magnet and was washed with ultrapure water and ethanol (20 mL, each). Afterwards, the reaction of the Fe_3_O_4_@SiO_2_ with 3-(trimethoxysilyl)propyl methacrylate (MPS) was carried out to provide Fe_3_O_4_@SiO_2_-MPS. An amount of 500 mg of the Fe_3_O_4_@SiO_2_ was suspended in a volume of 100 mL of anhydrous toluene and 10 mL of MPS in a three-neck, round-bottom flask. The mixture was allowed to react for 12 h under nitrogen. The Fe_3_O_4_@SiO_2_-MPS was separated again with a magnet and washed with ultrapure water (20 mL, each). The prepared particles were left in dry conditions for further synthesis of the molecularly imprinted shell.
